# SGLT2 Inhibitor–Associated Ketoacidosis vs Type 1 Diabetes–Associated Ketoacidosis

**DOI:** 10.1001/jamanetworkopen.2024.2744

**Published:** 2024-03-18

**Authors:** Mahesh M. Umapathysivam, Bethany Morgan, Joshua M. Inglis, Emily Meyer, Danny Liew, Venkatesan Thiruvenkatarajan, David Jesudason

**Affiliations:** 1 Southern Adelaide Diabetes and Endocrine Services, Flinders Medical Centre, Adelaide, South Australia, Australia; 2 Endocrine Department, Queen Elizabeth Hospital, Woodville, South Australia, Australia; 3 Endocrine Department, Royal Adelaide Hospital, Adelaide, South Australia, Australia; 4 Adelaide Medical School, University of Adelaide, Adelaide, South Australia, Australia; 5 Centre of Research Excellence in Translating Nutritional Science to Good Health, University of Adelaide, Adelaide, South Australia, Australia; 6 Anaesthetic Department, Queen Elizabeth Hospital, Woodville, South Australia, Australia; 7 Basil Hetzel Institute Woodville, South Australia, Australia

## Abstract

This cohort study examines the natural history and response to treatment of sodium glucose cotransporter 2 (SGLT2) inhibitor–associated ketoacidosis compared with that of type 1 diabetes–associated ketoacidosis.

## Introduction

The pathophysiology of sodium-glucose cotransporter 2 inhibitor (SGLT2i)–associated ketoacidosis (DKA) differs from type 1 diabetes (T1D) DKA. T1D DKA is driven by absolute insulin deficiency, leading to ketosis and hyperglycemia. In contrast, SGLT2i DKA occurs due to reduction in plasma glucose (PG) from urinary glucose losses, which reduces insulin secretion and stimulates glucagon secretion, leading to ketosis.^1,2,3^ Accordingly, PG levels in SGLT2i DKA are often normal or mildly elevated.^2,3^ The implication is that glycemia and ketosis are less closely linked than in T1D DKA. Despite these differences, the American Association of Clinical Endocrinologists and American College of Endocrinology recommends treatment with the same protocols as T1D.^4^ This may result in hypoglycemia when patients receive fixed-dose insulin infusion or inadequate insulin dosing and reduced ketone clearance when patients receive dynamic insulin infusions.

## Methods

We performed a retrospective cohort study to examine the natural history of SGLT2i DKA and its response to treatment. Ethics approval and a waiver of consent were provided by the Central Adelaide Local Health Network Human Research Research Ethics Committee per section 2.3.10 of the National Health and Medical Research Council National Statement. To assess the suitability of using T1D DKA protocols, particularly the dynamic insulin infusion protocol (eFigure in Supplement 1), in SGLT2i DKA, we compared ketone and bicarbonate level change after 24 hours of treatment in individuals presenting with SGLT2i DKA and T1D DKA.

Cases were identified from endocrine consultation requests on electronic medical record in 2 tertiary hospitals in South Australia between January 2019 and December 2021. All cases of SGLT2i DKA were among individuals using SGLT2is for type 2 diabetes. To reduce confounding by age, patients were included in the T1D group if they were 10 years younger than the mean age of the SGLT2i group; all patients in this age range were included. All patients in the SGLT2i group had SGLT2i therapy discontinued. If patients had multiple episodes of DKA, only the first was included in the analysis.

Both individuals with SGLT2i DKA (ketone >3 mmol/L; bicarbonate, <15 mEq/L) or clinically significant SGLT2i-associated ketosis (ketone, >3 mmol/L; bicarbonate, 15-20 mEq/L) were included in the SGLT2i group. To convert bicarbonate to millimoles per liter, multiply by 1.

Data were analyzed in April 2022. Data are presented as the median with IQR and were analyzed using a 2-sided Mann-Whitney test in PRISM version 10 (GraphPad). Multivariable linear regression was used to determine the factors associated with the rate of resolution in SGLT2i DKA as measured by ketone change over the first 24 hours of treatment. Variables were excluded in a backward stepwise manner (cutoff, *P* = .10). Analyses were undertaken in SPSS version 28.0 (IBM Corp). The variables included were preexisting insulin use, admission PG level, bicarbonate nadir, hemoglobin A_1c_ (HbA_1c_) level, insulin received in first 24 hours, duration of infusion, anion gap, body mass index, hematocrit level, age, and ketone peak. A 2-tailed *P* < .05 was considered statistically significant.

## Results

Thirty-seven episodes of SGLT2i DKA (n = 27) and SGLT2i-associated ketosis (n = 10) were identified, and 19 episodes of T1D DKA were identified in the specified age range. The groups were well matched for age and sex, but the SGLT2i group had a significantly higher body weight, lower HbA_1c_ levels, and significantly lower admission PG levels (Table).

**Table.** Baseline Characteristics of the SGLT2i and T1D Groups

Characteristic	Patients, median (IQR)		*P* value
	SGLT2i (n = 37)	T1D (n = 20)	
Sex, No. (%)			
Male	20 (54.1)	10 (50.0)	NA
Female	17 (45.9)	10 (50.0)	
Age, y	62 (57-70)	62 (54-74)	.84
Body mass index^a^	27.5 (24.2-32.0)	24.5 (23.5-28.5)	.14
Height, m	1.66 (1.61-1.72)	1.63 (1.55-1.70)	.34
Weight, kg	81.8 (65.0-91.3)	67.7 (54.3-80.8)	.04
Admission PG, mmol/L	12.5 (9.0-20.4)	36.8 (29.9-50.6)	<.001
Ketone peak, mmol/L	5.3 (4.3-5.9)	6.5 (4.7-7.0)	.02
Bicarbonate nadir, mEq/L	11.0 (6.0-15.0)	6.5 (5.8-11.0)	.12
HbA_1c_, mmol/mol	75 (55-95)	114 (100-143)	<.001
eGFR, mL/min/1.73 m^2^^b^	90 (90-90)	90 (80-90)	.02
Hypoglycemia in first 24 h of treatment, No. (%)	2 (5.4)	1 (5.9)	.99

Abbreviations: eGFR, estimated glomerular filtration rate; NA, not applicable; PG, plasma glucose; SGLT2i, sodium-glucose cotransporter 2; T1D, type 1 diabetes.

SI conversion: To convert bicarbonates to millimoles per liter, multiply by 1.

^a^ Body mass index calculated as weight in kilograms divided by height in meters squared.

^b^ If eGFR was greater than 90 mL/min/1.73 m^2^, it was included in analysis as 90. Data were available for 18 of 20 individuals in T1D group and all individuals in SGLT2i group.

Patients with SGLT2i DKA had milder DKA compared with T1D-DKA (median [IQR] ketone peak: 5.3 [4.2-5.9] vs 6.5 [4.7-7.2] mmol/L; *P* = .02). The SGLT2i group had delayed resolution (median (IQR) time: 36 [24-72] vs 18 [12-27] hours; *P* = .002) compared with the T1D group (Figure). Despite having a greater median (IQR) weight (81.8 [65.0-91.3] vs 67.7 [54.3-80.8] kg; *P* = .04), patients with SGLT2i DKA received a significantly lower insulin dose (intravenous and subcutaneous) in the first 24 hours of treatment compared with patients T1D DKA (median [IQR] dose: 44.0 [27.0-82.5] vs 87.0 [63.0-124.0] units; *P* = .01) (Figure).

**Figure.**
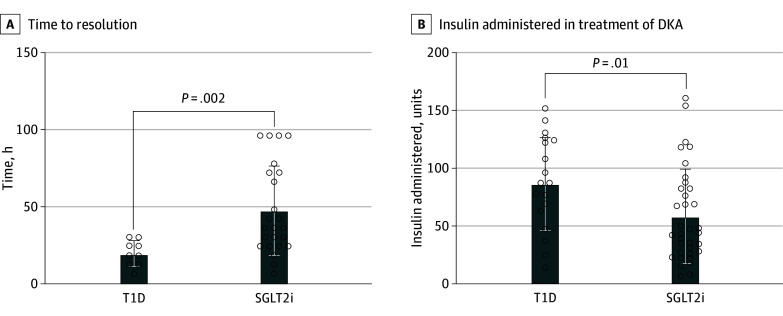
Time to Resolution of Ketosis and Acidosis and Amount of Insulin Administered in Patients With Type 1 Diabetes (T1D)–Associated and Sodium-Glucose Cotransporter 2 Inhibitor (SGLT2i)–Associated Diabetic Ketoacidosis (DKA) A, Individuals presenting with SGLT2i DKA (n = 24) had delayed time to resolution compared with age-matched individuals presenting with T1D-associated DKA (n = 9). B, Individuals with SGLT2i (n = 35) received less insulin in first 24 hours of treatment compared with age-matched individuals presenting with T1D-associated DKA (n = 19). The top of the bar shows the median values, and the whiskers show the IQR. Circles indicate individual values. The number of participants included in analysis varies as data were only included in time to resolution if analysis of both normalization of ketones and bicarbonate were recorded on electronic medical record.

The regression model was statistically significant (*R*^2^ = 0.68; *F*_(3, 17)_ = 11.901; *P* < .001). Change in ketone concentration over the first 24 hours was significantly associated with baseline insulin therapy (β = 6.67; 95% CI, 2.71 to 10.63; *P* = .002), lower bicarbonate nadir (β = −0.56; 95% CI, −0.89 to −0.23; *P* = .02), and higher admission PG level (β = 0.30; 95% CI, 0.04 to 0.55; *P* = .24) in SGLT2i DKA.

## Conclusions

In treatment of SGLT2i DKA, a tendency toward euglycemia results in clinically relevant reduction in the amount of insulin administered over the first 24 hours of treatment. It remains unclear whether the protracted duration of SGLT2i DKA is a result of inadequate insulin dosing or the ongoing effect of SGLT2i. It would be reasonable, based on the evidence presented and the safety profile of intravenous dextrose, to increase dextrose infusion rates and concentration to allow increased insulin administration and suppression of ketosis. The limitations of this study are its retrospective nature and size. Prospective randomized clinical trial evidence is lacking.

## Notes

Supplement 1. **eFigure.** Insulin Infusion Algorithm for Diabetic Ketoacidosis

Supplement 2.
Data Sharing Statement
