# Supplementary material for: SGLT2 Inhibitor–Associated Ketoacidosis vs Type 1 Diabetes–Associated Ketoacidosis
Source: JAMA Netw Open. 2024 Mar 18;7(3):e242744. doi: 10.1001/jamanetworkopen.2024.2744 (PMC10949093; doi:10.1001/jamanetworkopen.2024.2744)
Supplement: Supplement 1. — eFigure. Insulin Infusion Algorithm for Diabetic Ketoacidosis [file jamanetwopen-e242744-s001.pdf]

Supplemental Online Content

Umapathysivam MM, Morgan B, Inglis JM, et al. SGLT2 inhibitor–associated ketoacidosis vs type 1 diabetes–associated ketoacidosis. *JAMA Netw Open.* 2024;7(3):e242744. doi:10.1001/jamanetworkopen.2024.2744

eFigure. Insulin Infusion Algorithm for Diabetic Ketoacidosis

This supplemental material has been provided by the authors to give readers additional information about their work.

© 2024 Umapathysivam MM et al. *JAMA Network Open.*

eFigure. Insulin Infusion Algorithm for Diabetic Ketoacidosis

CALHN

☐ TQEH ☐ RAH

MEDICATION CHART
ACTRAPID INSULIN INFUSION ALGORITHM
DIABETIC KETOACIDOSIS

USE **EPAS** PATIENT IDENTIFICATION LABEL

Unit Record Number: ____________

Do not hand write these details, except when adhesive barcode labels are unavailable

Visit Number: ____________

Surname: ____________

Given Names: ____________

Date of Birth: ____________ Sex: ____________

This form is to be used with a medical order in EPAS for Actrapid insulin infusion for the management of DIABETIC KETOACIDOSIS

- Use the Actrapid insulin infusion algorithm below to titrate the insulin dose according to the patient's blood glucose level.
- This form remains in the patient's temporary folder while the Actrapid insulin infusion continues.
- On completion of the Actrapid insulin infusion scan form into EPAS against the admission visit.
- A new form is to be used with each new commencement of an Actrapid insulin infusion.

For Diabetic Ketoacidosis (DKA) management guidelines and EPAS ordering instructions refer to:
CALHN-OWI04404 Diabetes: Diabetes Ketoacidosis (DKA) Management - (including Insulin Infusion and Intravenous Therapy).

ACTRAPID INSULIN INFUSION ALGORITHM Commencement date: ___/___/ 20___ **time:** _____ **hours**

COMMENCING INFUSION:

Always start in Column 1 when initially commencing infusion

OR when recommencing infusion after it has been turned off for an hour or more

OR when recommencing infusion after a hypoglycaemic episode

Adjusting Insulin Infusion:

If BGL 5.0-10.0mmol/L (in target) → Stay in the same column

If BGL is greater than 10.0mmol/L
- and BGL has decreased by 2.0mmol/L or more since last measurement → Stay in the same column
- and BGL has decreased by less than 2.0mmol/L OR increased since last measurement → Move from **Column 1** to **Column 2** OR stay in **Column 2**

Turn Insulin Infusion off if:
- BGL is less than 5mmol/L (check BGL in 1 hour)

OR
- Patient has hypoglycaemia (follow hypoglycaemia protocol)

INSULIN INFUSION ALGORITHM
Target BGL range: 5 – 10mmol/L

BGL less than 4.0mmol/L suggests hypoglycaemia. Follow hypoglycaemia protocol.		BGL less than 4.0mmol/L suggests hypoglycaemia. Follow hypoglycaemia protocol.	
**Column 1**		**Column 2** (higher dose)	
**BGL mmol/L**	**Units/hr**	**BGL mmol/L**	**Units/hr**
Less than 5.0	Off	Less than 5.0	Off
5.0 – 7.9	0.5	5.0 – 7.9	1
8.0 – 9.9	1	8.0 – 9.9	2
10.0 – 11.4	1.5	10.0 – 11.4	3
11.5 – 12.9	2	11.5 – 12.9	4
13.0 – 14.9	3	13.0 – 14.9	5
15.0 – 16.4	3	15.0 – 16.4	6
16.5 – 17.9	4	16.5 – 17.9	7
18.0 – 20.0	5	18.0 – 20.0	8
Greater than 20.0	6	Greater than 20.0	12

If BGL is greater than 15mmol/L for 3 consecutive hours or greater than 12mmol/L for 6 consecutive hours contact doctor for review

If insulin infusion running at 10units/hour or more and BGL remains elevated consider malfunction of system e.g. non-patent infusion pump, blood glucose meter malfunction, and non-patent IV access.

Medication

March 2018

DKA ACTRAPID INSULIN INFUSION

MR 94.2(a)

© 2024 Umapathysivam MM et al. *JAMA Network Open.*
