# Supplementary material for: SGLT2 Inhibitor–Associated Ketoacidosis vs Type 1 Diabetes–Associated Ketoacidosis
Source: JAMA Netw Open. 2024 Mar 18;7(3):e242744. doi: 10.1001/jamanetworkopen.2024.2744 (PMC10949093; doi:10.1001/jamanetworkopen.2024.2744)
Supplement: Supplement 2. — Data Sharing Statement [file jamanetwopen-e242744-s002.pdf]

Data Sharing Statement

Umapathysivam. SGLT2 Inhibitor–Associated Ketoacidosis vs Type 1 Diabetes–Associated Ketoacidosis. *JAMA Netw Open*. Published March 18, 2024.
doi:10.1001/jamanetworkopen.2024.2744

Data
Data available: No
